# MRI software for diffusion-perfusion mismatch analysis may impact on patients’ selection and clinical outcome

**DOI:** 10.1007/s00330-021-08211-2

**Published:** 2021-08-05

**Authors:** Silvia Pistocchi, Davide Strambo, Bruno Bartolini, Philippe Maeder, Reto Meuli, Patrik Michel, Vincent Dunet

**Affiliations:** 1 Diagnostic Neuroradiological Unit, Service of Diagnostic and Interventional Radiology, Department of Medical Radiology, Lausanne University Hospital and University of Lausanne, Lausanne, Switzerland; 2 Stroke Center, Service of Neurology, Department of Clinical Neurosciences, Lausanne University Hospital and University of Lausanne, Lausanne, Switzerland

**Keywords:** Stroke, Thrombectomy, Software, Diffusion, Perfusion

## Abstract

**Objective:**

Impact of different MR perfusion software on selection and outcome of patients with acute ischemic stroke (AIS) and large vessel occlusion (LVO) treated by endovascular thrombectomy (EVT) is unclear. We aimed at comparing two commercial MRI software, semi-automated with unadjusted (method A) and adjusted mask (method B), and fully automated (method C) in this setting.

**Methods:**

MRI from 144 consecutive AIS patients with anterior circulation LVO was retrospectively analysed. All diffusion- and perfusion-weighted images (DWI-PWI) were post-processed with the three methods using standard thresholds. Concordance for core and hypoperfusion volumes was assessed with Lin’s test. Clinical outcome was compared between groups in patients who underwent successful EVT in the early and late time window.

**Results:**

Mean core volume was higher and mean hypoperfusion volume was lower in method C than in methods A and B. In the early time window, methods A and B found fewer patients with a mismatch ratio ≤ 1.2 than method C (1/67 [1.5%] vs. 12/67 [17.9%], *p* = 0.0013). In the late time window, methods A and B found fewer patients with a mismatch ratio < 1.8 than method C (3/46 [6.5%] and 2/46 [4.3%] vs. 18/46 [39.1%], *p* ≤ 0.0002). More patients with functional independence at 3 months would not have been treated using method C versus methods A and B in the early (*p* = 0.0063) and late (*p* ≤ 0.011) time window.

**Conclusions:**

MRI software for DWI-PWI analysis may influence patients’ selection before EVT and clinical outcome.

**Key Points:**

• *Method C detects fewer patients with favourable mismatch profile.*

• *Method C might underselect more patients with functional independence at 3 months.*

• *Software used before thrombectomy may influence patients’ outcome.*

## Introduction

Endovascular therapy for patients with acute ischemic stroke (AIS) is nowadays supported by class A evidence [1–6]. Beyond 6 h from last proof of good health (LPGH), AIS patient with large vessel occlusion (LVO) might still benefit from endovascular therapy when they met either a clinical-core mismatch or core-hypoperfusion mismatch (i.e. mismatch ratio ≥ 1.8) [7, 8], using severely ischemic tissue with uncertain viability (SIT-uv) imaging (called ‘core’ in this manuscript) as a base [9]. As a consequence, perfusion CT or MR imaging is now the standard of care for patients presenting with acute stroke [10]. Many stroke centres introduced automated perfusion processing software in their clinical practice in order to save time and to reduce inter-observer variability [11]. However, it has been proven that some tools require improvement in order to be able to reliably differentiate between patients with a credible ischemia-related region of hypoperfusion and those without [12].

Current literature on AIS perfusion imaging is mostly based on CT imaging. A comprehensive analysis on CT perfusion imaging [13] has shown that there is marked variability in penumbra and infarct prediction among various deconvolution techniques. Xiong et al [14] compared two automated CT perfusion software (RAPID® and Olea Sphere®) for evaluation of AIS patients, showing that core volumes calculated with RAPID® were larger than with Olea Sphere®. The only report about MR perfusion software actually available, recently published by Deutschmann et al [15], compared RAPID® and Olea Sphere® software. They reported a small systematic difference between software, in the sense that RAPID® outlines slightly larger ADC and smaller hypoperfused tissue volumes. In this context, it seems straightforward to suppose that different perfusion software perform differently in AIS patients’ classification. To the best of our knowledge, there is no study that compared RAPID® and Carestream® MR perfusion software and none that evaluated the potential impact on patients’ outcome.

In the present study, we aimed at evaluating the concordance between these two software for core and hypoperfusion volumes’ estimation in AIS patients with anterior circulation LVO. The second endpoint was to assess the potential impact of volumes’ discrepancies on patients’ classification prior therapy and on patients’ functional independence at 3 months.

## Materials and methods

### Patient cohort

All consecutive patients with acute ischemic stroke and occlusion of ICA or MCA (M1 or M2 segment) who underwent an acute MR imaging between May 2018 and June 2020 at our institution were retrospectively extracted from the Acute STroke Registry and Analysis of Lausanne (ASTRAL) (*N* = 178). ASTRAL collects all acute ischemic strokes admitted to the stroke unit and intensive care unit of the Lausanne University Hospital presenting within 24 h of stroke onset or LPGH [16].

In this cohort, the inclusion criterion was availability of both DWI and PWI raw data (*N* = 144/178, 81%); MRI with movement artefacts was also included to compare performance of the two software in ‘real life’ setting. There were no additional exclusion criteria.

Clinical and epidemiological data, including occlusion site, sex, age, cardiovascular risk factors, aetiology classified according to the Trial of Org 10172 in Acute Stroke Treatment (TOAST) criteria [17], National Institutes of Health Stroke Scale (NIHSS) and treatment type, including intravenous tissue plasminogen activator (IV tPA) and/or EVT, were prospectively collected and extracted from ASTRAL. Eligibility for endovascular treatment followed current guidelines. In the early time window, the presence of acute stroke symptoms and anterior circulation LVO was a sufficient criterion to undergo an EVT. In the late time window, we used liberal clinical/imaging mismatch criteria, based on the NIHSS and DWI-ASPECTS, as previously reported [18]. Treatment decision was thus made independently of any software result in order to avoid potential software-related selection bias. Primary revascularisation following EVT was assessed with the modified Treatment in Cerebral Ischemia (mTICI) score. A successful revascularisation was defined as mTICI superior or equal to 2b. Outcome, expressed as NIHSS at 7 days and modified Rankin score (mRS) at 7 days and at 3 months, and symptomatic intracranial haemorrhage according to the criteria of the European Cooperative Acute Stroke Study (sICHECASS) were also extracted from ASTRAL. A favourable outcome was defined as functional independence (i.e. mRS = 0 to 2) [19]. The ethics committee for research on humans of the Canton of Vaud approved collection, analysis and publication of data from ASTRAL, without requesting patients’ consent.

### Imaging acquisition and post-processing

All MR examinations were performed on a MAGNETOM VIDA 3T system (Siemens Healthcare) in the Emergency Radiology Unit of our institution, according to the standard protocol used in our department for AIS.

MRI was performed with a 64-channel head coil and included the following sequences: sagittal T1-weighted (TR/TE: 400.0 ms/2.46 ms, slice thickness 3 mm, FoV 250 mm), axial diffusion imaging (TR/TE: 3000.0 ms/80.00 ms, slice thickness 3 mm, FoV 239 mm), axial fluid-attenuated inversion recovery (TR/TE: 9000.0 ms/87.00 ms, slice thickness 3 mm, FoV 230 mm), axial T2*-weighted gradient echo (TR/TE: 1070.0 ms/19.80 ms, slice thickness 3 mm, FoV 230 mm), axial 3D TOF angiography (TR/TE: 21.0 ms/3.69 ms, slice thickness 0.5 mm, FoV 220 mm), DSC perfusion axial imaging (TE/TR: 2430.0 ms/25.00 ms, slice thickness 3 mm, FoV 215 mm) and axial T1-weighted post-contrast media (TE/TR: 400.0 ms/2.61 ms, slice thickness 3 mm, FoV 230 mm). Gadolinium-based contrast media (Dotarem, Guerbet) are administered intravenously via a power injection with a dose of 0.1 mmol/kg body weight at 4 mL/s flow followed by 20 mL of NaCl 0.9% at the same flow, as recommended by ASFNR guidelines [20].

DWI and PWI were processed with two commercial software in three different methods. In all cases, the same thresholds were applied. The core was defined as an apparent diffusion coefficient (ADC) value inferior to 620 × 10^−6^ mm^2^/s [21] and the hypoperfusion as a *T*_max_ superior to 6 s [22, 23]. The mismatch ratio was calculated as hypoperfusion/core.

#### Method A

Carestream® Picture Archiving and Communication System (PACS) (Version 12.2.2.1025, Philips Healthcare Information Solutions) incorporates stroke perfusion software analysis. Once DWI-PWI raw data arrive on the PACS, the radiologist activates the reconstruction using the ‘Perfusion Stroke’ module and then chooses the affected hemisphere. No further manipulations of the brain mask are applied for this analysis. This version provides perfusion parameter maps (cerebral blood flow, cerebral blood volume, mean transit time and *T*_max_) and ADC map. Volumes of core, and hypoperfusion, are calculated in millilitres using thresholds set as previously described, and saved in a table on the PACS. The mismatch ratio (hypoperfusion/core) is manually calculated.

#### Method B

**Fig. 1:**
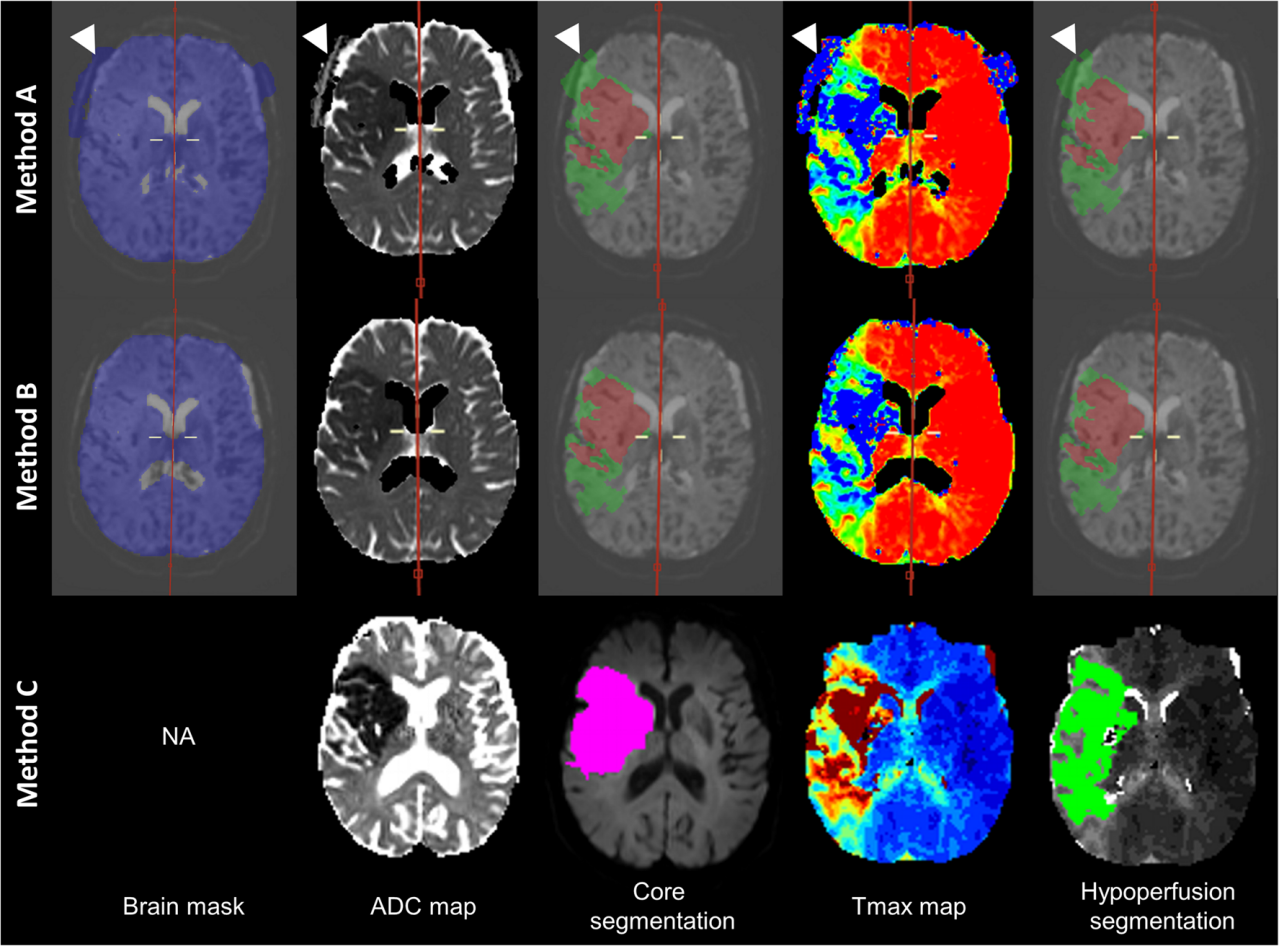
Exemplary case of volumes’ segmentation with the three methods. Case of a 77-year-old female with right M1 segment occlusion diagnosed in the early time window. The core volume, hypoperfusion volume and mismatch ratio were estimated as 47 mL, 70 mL and 1.5 (i.e. favourable mismatch profile) with method A (top line); 47 mL, 65 mL and 1.4 (i.e. favourable mismatch profile) with method B (midline); and 93 mL, 100 mL and 1.1 (i.e. unfavourable mismatch profile) with method C (bottom line). White arrows indicate brain mask error found on method A that was removed on method B. The patient underwent an early EVT and had moderate disability at 3 months (mRS = 3)

Same steps described in method A are followed with additional manual adjustment of the brain mask, which allows suppression of extracerebral mask errors (i.e. suppression of area outside the brain volume) when necessary and is part of standard options of the ‘Perfusion Stroke’ tool. Core and hypoperfusion volumes are then estimated the same way as described for method A. For the purpose of this study, two neuroradiologists (S.P. and V.D. with 10 years’ experience in neuroradiology) performed the manual brain mask adjustment of the cohort in consensus reading. This procedure has been performed for all cases and took two additional minutes per case. An exemplary case is shown in Fig. 1.

#### Method C

RAPID® (iSchemaView) fully automatically analysed raw data received from MR system and sends the results to our PACS, reporting core and hypoperfusion volumes in millilitres as well as mismatch ratio on a single summary image.

**Fig. 2:**
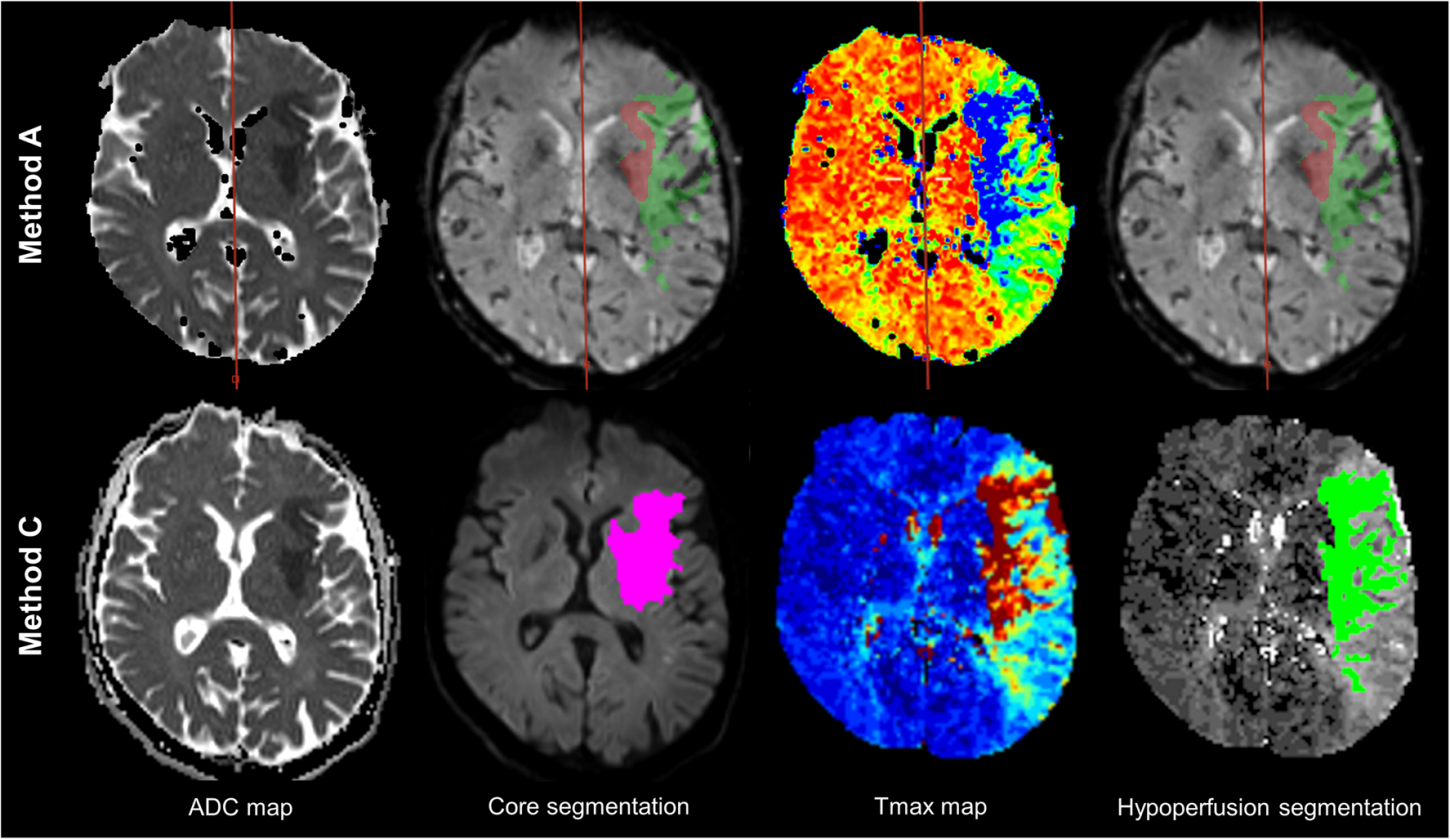
Exemplary case of volumes’ segmentation. Case of a 59-year-old man with left M2 segment occlusion diagnosed in the late time window. The core volume, hypoperfusion volume and mismatch ratio were estimated as 15.2 mL, 66.4 mL and 4.4 (i.e. favourable mismatch profile) with methods A (top line) and B, and 38 mL, 65 mL and 1.7 (i.e. unfavourable mismatch profile) with method C (bottom line). The patient underwent a late EVT and had functional independence at 3 months (mRS = 2)

For every method, the core and hypoperfusion volumes were recorded. The DWI-PWI mismatch ratio was recorded in a dichotomous way. In the early time window, a mismatch ratio > 1.2 defined a favourable profile, while a mismatch ratio ≤ 1.2 defined an unfavourable profile, according to the FRAME analysis [3, 24]. In the late time window, a mismatch ratio ≥ 1.8 defined a favourable profile, while a ratio < 1.8 defined an unfavourable profile, according to DEFUSE 3 study criteria. An exemplary case is shown in Fig. 2.

### Statistical analysis

All statistics were performed with the Stata software (version 15.0, StataCorp). Continuous variables are presented as mean ± standard deviation and categorical variables as number or percentage. For further analysis, we divided our study population according to the time window from LPGH to imaging (early ≤ 6 h or late > 6 h). Continuous variables and categorical variables were compared between groups using the Wilcoxon signed-rank test or Fisher exact test. Inter-method concordance for core and hypoperfusion volume estimation was assessed by Lin’s test with estimation of the Pearson correlation coefficient (rho), concordance correlation coefficient (rho_c), bias correction factor (C_b) and calculation of mean difference with 95% Bland-Altman limits-of-agreement (95% LOA). Agreement for mismatch-based patients’ classification was assessed with Gwet’s AC1 (95%CI). Correlation coefficient and Gwet’s AC1 were interpretated according to the modified Landis and Koch scale as follows: poor when < 0.00, slight between 0.00 and 0.20, fair between 0.21 and 0.40, moderate between 0.41 and 0.60, good between 0.61 and 0.80 and excellent above 0.81. Finally, for each method, clinical outcome (NIHSS at 7 days, mRS at 7 days and 3 months) was compared between classification groups in patients who underwent EVT in the early and late windows. A *p* value < 0.05 was considered statistically significant. Adjustment of the significance level was done according to the Bonferroni method for every multiple comparisons presented in Tables 4 and 5.

## Results

### Study population and technical success of perfusion imaging

Demographic data and clinical information for the 144 enrolled patients are provided in Table 1. Out of 144, 74 (74/144, 51.4%) were treated with intravenous thrombolysis, 113 (113/144, 78.4%) with EVT and 60 (60/144, 41.7%) with both in ‘bridging’ technique. Of 113 patients undergoing EVT, 67 were treated in the early time window and 46 patients in the late time window.

**Table 1:** Population characteristics

Enrolled patients	*N* = 144
Males	71 (49.3%)
Age (y)	74.6 ± 11.8
Cardiovascular risk factors	
Hypertension	112 (77.7%)
Diabetes	37 (25.7%)
Hypercholesterolemia	114 (79.2%)
Smoking	38 (26.4%)
Atrial fibrillation	58 (40.3%)
Coronaropathy	36 (25%)
Active cancer	16 (11.1%)
Stroke aetiology (TOAST)	
Large artery atherosclerosis	22 (15.3%)
Cardiac embolism	65 (45.1%)
Small vessel disease	0 (0%)
Cervical artery dissection	3 (2%)
Patent foramen ovale	0 (0%)
Other determined aetiologies	10 (6.9%)
More than one possible aetiology	10 (6.9%)
Unknown aetiology despite complete evaluation	22 (15.3%)
Unknown aetiology with incomplete evaluation	6 (4.2%)
NIHSS at admission	12.4 ± 7.2
Treatment	
IVT	74 (51.4%)
EVT	115 (79.9%)
Conservative	15 (10.4%)
Outcome	
NIHSS at 7 days	9.7 ± 13.3
mRS at 7 days	3.1 ± 1.6
mRS 3 months	3.2 ± 2.0
sICHECASS	5 (3.5%)

DWI-PWI analysis was successfully performed with methods A and B in all 144 patients and in 142/144 patients (99%) with method C. In two cases, method C failed to perform the post-processing: in the first case, it did not calculate both core and hypoperfusion volumes, and in the second case, it did not calculate hypoperfusion volume. We excluded these two patients from further analysis.

### Inter-method concordance analysis

Estimated absolute mean values of core and hypoperfusion volumes as well as patients’ classification based on dichotomised mismatch ratio are shown in Table 2.

**Table 2:** Estimated volumes and patients’ classification

Parameters	Method A	Method B	Method C
All patients (*N* = 142)			
Core (mL)	22.7 ± 38.7	19.1 ± 35.3	36.8 ± 52.2
Hypoperfusion (mL)	160.3 ± 118.9	117.9 ± 78.4	84.5 ± 74.0
Early EVT (*N* = 67)			
Core (mL)	19.2 ± 34.7	15.8 ± 31.5	31.0 ± 46.1
Hypoperfusion (mL)	166.8 ± 122.7	121.2 ± 71.1	82.9 ± 60.1
Mismatch ratio			
≤ 1.2	1 (1.5%)	1 (1.5%)	12 (17.9%)
> 1.2	66 (98.5%)	66 (98.5%)	55 (82.1%)
Late EVT (*N* = 46)			
Core (mL)	20.4 ± 25.7	17.4 ± 24.4	35.6 ± 44.5
Hypoperfusion (mL)	144.1 ± 106.4	111.1 ± 78.6	83.3 ± 77.8
Mismatch ratio			
< 1.8	3 (6.5%)	2 (4.3%)	18 (39.1%)
≥ 1.8	43 (93.5%)	44 (95.7%)	28 (60.9%)

Mismatch ratio cutoff values followed EXTEND-IA criteria in the early time window and DEFUSE 3 criteria in the late time window. *EVT* endovascular thrombectomy

Statistical agreement analysis is shown in Table 3. Regarding core volume, methods A and B had an excellent concordance. Compared to method A or B, the core volume was larger using method C despite an overall good concordance between methods. Regarding hypoperfusion volume, methods A and B had a good concordance. Compared to methods A and B, the hypoperfusion volume was lower using method C, with a fair to moderate concordance.

**Table 3:** Inter-method volumes’ concordance

Pairs	Pearson’ rho	Rho_c	C_b	Mean difference	95% LOA
All patients (*N* = 142)					
*Core volume*					
Method A versus B	0.97	0.96	0.99	3.6 ± 10.0	−16.0 to 23.1
Method A versus C	0.90	0.82	0.91	−14.1 ± 24.5	−62.2 to 34.0
Method B versus C	0.94	0.80	0.86	−18.1 ± 23.7	−64.5 to 28.2
*Hypoperfusion volume*					
Method A versus B	0.74	0.62	0.85	41.8 ± 80.5	−115.9 to 199.6
Method A versus C	0.38	0.27	0.69	75.8 ± 113.4	−146.5 to 298.2
Method B versus C	0.56	0.51	0.91	33.4 ± 71.7	−107.1 to 174.0
Early EVT (*N* = 67)					
*Core volume*					
Method A versus B	0.97	0.96	0.99	3.4 ± 8.9	−14.2 to 20.9
Method A versus C	0.88	0.81	0.92	−11.8 ± 22.9	−56.8 to 33.1
Method B versus C	0.92	0.79	0.87	−15.2 ± 21.5	−57.2 to 26.8
*Hypoperfusion volume*					
Method A versus B	0.69	0.54	0.79	45.6 ± 89.7	−130.2 to 221.4
Method A versus C	0.37	0.21	0.57	83.8 ± 115.0	−141.5 to 309.2
Method B versus C	0.59	0.50	0.84	38.2 ± 60.0	−79.4 to 155.9
Late EVT (*N* = 46)					
*Core volume*					
Method A versus B	0.97	0.96	0.99	3.0 ± 6.5	−9.8 to 15.8
Method A versus C	0.93	0.74	0.80	−15.2 ± 22.5	−59.3 to 28.8
Method B versus C	0.94	0.70	0.75	−18.2 ± 23.2	−63.7 to 27.2
*Hypoperfusion volume*					
Method A versus B	0.88	0.79	0.90	33.0 ± 52.4	−69.6 to 135.7
Method A versus C	0.51	0.40	0.78	60.8 ± 94.6	−124.6 to 246.2
Method B versus C	0.63	0.60	0.94	27.8 ± 67.0	−103.5 to 159.0

*EVT* endovascular thrombectomy, *LOA* limits-of-agreement

### Estimation of unfavourable mismatch profile and outcome in patients who underwent EVT

#### Early time window

In the early time window, of the 67 patients who underwent EVT, 63 (94%) had successful recanalisation (mTICI = 2b–3). Methods A and B found a mismatch ratio ≤ 1.2 in 1/67 (1.5%), and method C in 12/67 (17.9%) patients. Method C thus indicated more unfavourable mismatch profile (*p* = 0.0013). The agreement to identify a mismatch ratio ≤ 1.2 vs. > 1.2 was excellent between methods A and B (Gwet’s AC1: 0.97 [0.92–1.0]) and only good between method C and methods A and B (Gwet’s AC1: 0.76 [0.62–0.90]). Regarding patients’ outcome for these two subgroups, only one patient was classified as having an unfavourable mismatch profile with methods A and B, and had a poor outcome despite successful recanalization. All of the 31/63 (49.2%) patients who were functionally independent at 7 days and 3 months after successful EVT had a favourable mismatch profile according to methods A and B. Compared to patients with an unfavourable mismatch profile, the proportion of patients with a favourable mRS at 7 days and 3 months was higher in the case of a favourable mismatch profile as defined by methods A, B and C. However, 6/31 (19.4%) patients with a favourable outcome would not have been treated when only taking into account the mismatch profile attributed by method C (versus 0% with methods A and B, *p* = 0.0063). All outcome data are reported in Table 4.

**Table 4:** EVT patients’ outcome in the early time window

Method A	TICI2b/2c/3, *n* = 63	MR ≤ 1.2, *n* = 1	MR > 1.2, *n* = 62	*p* value
NIHSS admission NIHSS 7d	12.3 ± 7.3 7.4 ± 12.2^‡^	27 28	12.1 ± 7.1 7.0 ± 12.0^‡^	NA NA
mRS pre-stroke mRS 7d mRS 3 months mRS 0–2 at 7d mRS 0–2 at 3m	1.0 ± 1.1 2.8 ± 1.6 2.7 ± 1.9 31/63 (49.2%) 31/63 (49.2%)	0 5 3 0/63 (0%) 0/63 (0%)	1.1 ± 1.1 2.7 ± 1.6 2.7 ± 1.9 31/63 (49.2%) 31/63 (49.2%)	NA NA NA < 0.0001 < 0.0001
sICHECASS	3/63 (4.8%)	0/63 (0%)	3/63 (4.8%)	0.07
Method B	TICI2b/2c/3, *n* = 63	MR ≤ 1.2, *n* = 1	MR > 1.2, *n* = 62	*p* value
NIHSS admission NIHSS 7d	12.3 ± 7.3 7.4 ± 12.2^‡^	20 42	12.2 ± 7.3 6.8 ± 11.4^‡^	NA NA
mRS pre-stroke mRS 7d mRS 3 months mRS 0–2 at 7d mRS 0–2 at 3m	1.0 ± 1.1 2.8 ± 1.6 2.7 ± 1.9 31/63 (49.2%) 31/63 (49.2%)	0 6 6 0/63 (0%) 0/63 (0%)	1.1 ± 1.1 2.7 ± 1.5 2.6 ± 1.9 31/63 (49.2%) 31/63 (49.2%)	NA NA NA < 0.0001 < 0.0001
sICHECASS	3/63 (4.8%)	0/63 (0%)	3/63 (4.8%)	0.07
Method C	TICI2b/2c/3, *n* = 63	MR ≤ 1.2, *n* = 12	MR > 1.2, *n* = 51	*p* value
NIHSS admission NIHSS 7d	12.3 ± 7.3 7.4 ± 12.2^‡^	13.2 ± 8.0 6.6 ± 7.7	12.1 ± 7.1 7.6 ± 13.2^†^	0.62 0.48
mRS pre-stroke mRS 7d mRS 3 months mRS 0–2 at 7d mRS 0–2 at 3m	1.0 ± 1.1 2.8 ± 1.6 2.7 ± 1.9 31/63 (49.2%) 31/63 (49.2%)	1.0 ± 0.9 2.8 ± 1.6 2.6 ± 2.1 6/63 (9.5%) 6/63 (9.5%)	1.1 ± 1.2 2.8 ± 1.6 2.7 ± 1.9 25/63 (39.7%) 25/63 (39.7%)	0.98 0.21 0.23 < 0.0001 < 0.0001
sICHECASS	3/63 (4.8%)	0/63 (0%)	3/63 (4.8%)	0.07

*EVT* endovascular thrombectomy, *MR* mismatch ratio, *mRS* modified Rankin scale, *NIHSS* National Institutes of Health Stroke Scale, *TICI* Thrombolysis in Cerebral Infarction. *p* values in the right column referred to comparison between favourable and unfavourable mismatch profiles. *p* value < 0.05, _corr_*p* value < 0.005. ^†^*p* < 0.05 for NIHSS at 7 days compared to NIHSS at admission. ^‡^*p* < 0.005 for NIHSS at 7 days compared to NIHSS at admission

#### Late time window

In the late time window, of the 46 patients who underwent EVT, 42 (91.3%) had successful recanalization. Method A indicated a mismatch ratio < 1.8 in 3/46 (6.5%) patients, method B in 2/46 (4.3%) patients and method C in 18/46 (39.1%) patients (*p* ≤ 0.0002). The agreement was excellent between methods A and B (Gwet’s AC1: 0.93 [0.84–1.0]), but it was only moderate between method C and methods A and B (Gwet’s AC1: 0.50 [0.22–0.77] and 0.47 [0.20–0.75], respectively). Compared to patients with an unfavourable mismatch profile, the proportion of patients with a favourable mRS at 7 days and 3 months was higher in the case of a favourable mismatch profile as defined by methods A and B (*p* ≤ 0.0002). On the contrary, the proportion of patients with a favourable mRS at 7 days and 3 months was similar between patients with an unfavourable and a favourable mismatch profile defined by method C (*p* ≥ 0.014). Out of 18 patients having a good mRS at 3 months, 7 (38.9%) would not have been treated taking into account the profile attributed by method C versus 3 (16.6%) and 1 (5.5%) using methods A and B (*p* = 0.011 and *p* < 0.0001, respectively). All outcome data are reported in Table 5.

**Table 5:** EVT patients’ outcome in the late time window

Method A	TICI2b/3, *n* = 42	MR < 1.8, *n* = 3	MR ≥ 1.8, *n* = 39	*p* value
NIHSS admission NIHSS 7d	13.8 ± 6.3 8.6 ± 11.0^†^	9.3 ± 3.1 3.0 ± 2.6^†^	14.2 ± 6.4 9.1 ± 11.4^†^	0.14 0.58
mRS pre-stroke mRS 7d mRS 3 months mRS 0–2 at 7d mRS 0–2 at 3m	1.1 ± 1.1 3.0 ± 1.4 3.0 ± 1.9 15/42 (35.7%) 18/42 (42.9%)	1.3 ± 0.6 2.3 ± 0.6 1.7 ± 0.6 2/42 (4.8%) 3/42 (7.1%)	1.1 ± 1.1 3.1 ± 1.5 3.1 ± 1.9 13/42 (31.0%) 15/42 (35.7%)	0.54 0.36 0.21 0.0002 0.0001
sICHECASS	2/42 (4.8%)	0/42 (0%)	2/42 (4.8%)	0.27
Method B	TICI2b/3, *n* = 42	MR < 1.8, *n* = 2	MR ≥ 1.8, *n* = 40	*p* value
NIHSS admission NIHSS 7d	13.8 ± 6.3 8.6 ± 11.0^†^	11.0 ± 1.4 18.5 ± 24.7	14.0 ± 6.4 8.1 ± 10.2^‡^	0.46 0.57
mRS pre-stroke mRS 7d mRS 3 months mRS 0–2 at 7d mRS 0–2 at 3m	1.1 ± 1.1 3.0 ± 1.4 3.0 ± 1.9 15/42 (35.7%) 18/42 (42.9%)	1.0 ± 0.0 3.5 ± 2.1 3.5 ± 3.5 1/42 (2.4%) 1/42(2.4%)	1.2 ± 1.1 3.0 ± 1.4 2.9 ± 1.8 14/42 (33.3%) 17/42 (40.5%)	1.0 0.63 0.94 < 0.0001 < 0.0001
sICHECASS	2/42 (4.8%)	0/42 (0%)	2/42 (4.8%)	0.27
Method C	TICI2b/3, *n* = 42	MR < 1.8, *n* = 16	MR ≥ 1.8, *n* = 26	*p* value
NIHSS admission NIHSS 7d	13.8 ± 6.3 8.6 ± 11.0^†^	14.9 ± 6.1 9.3 ± 9.8^†^	13.2 ± 6.4 8.1 ± 12.1^†^	0.53 0.28
mRS pre-stroke mRS 7d mRS 3 months mRS 0–2 at 7d mRS 0–2 at 3m	1.1 ± 1.1 3.0 ± 1.4 3.0 ± 1.9 15/42 (35.7%) 18/42 (42.9%)	1.3 ± 0.8 3.2 ± 1.1 2.9 ± 1.7 4/42 (9.5%) 7/42 (16.7%)	1.0 ± 1.2 2.9 ± 1.6 3.0 ± 2.1 11/42 (26.2%) 11/42 (26.2%)	0.18 0.46 0.88 0.014 0.16
sICHECASS	2/42 (4.8%)	1/42 (2.4%)	1/42 (2.4%)	1.0

*EVT* endovascular thrombectomy, *MR* mismatch ratio, *mRS* modified Rankin scale, *NIHSS* National Institutes of Health Stroke Scale, *TICI* Thrombolysis in Cerebral Infarction. *p* values in the right column referred to comparison between favourable and unfavourable mismatch profiles. *p* value < 0.05, _corr_*p* value < 0.005. ^†^*p* < 0.05 for NIHSS at 7 days compared to NIHSS at admission. ^‡^*p* < 0.005 for NIHSS at 7 days compared to NIHSS at admission

## Discussion

Three main results from the present study are as follows: (1) despite using identical core/penumbra thresholds, core volumes are larger and hypoperfusion volumes are smaller when estimated by method C; (2) this results in a higher proportion of patients with unfavourable DWI-PWI mismatch profile using method C, both in the early and late time windows; (3) in patients with functional independence at 3 months after successful late EVT, method C would have underselected more patients than methods A and B. Overall the analysis of outcome of patients that underwent successful EVT suggests that some DWI-PWI software may be too restrictive.

MRI is considered the gold standard in the detection of AIS. Nevertheless, unlike CT, MRI is not widely available in acute stroke settings. Accordingly, current literature on AIS perfusion imaging based on MR imaging modality is still scarce.

In this study, we compared two commercially available MR perfusion software using three methods. We found larger core volume and lower hypoperfusion volume using method C compared to methods A and B. This observation was made for AIS patients with anterior circulation LVO admitted either in the early or in late time window who underwent EVT. This is in line with the study by Deutschmann et al [15] who compared RAPID® and Olea Sphere® software and reported that RAPID® outlines slightly larger ADC and smaller hypoperfused tissue volumes. They considered that small differences of the outlined regions in single slices can sum up to considerable differences in 3D volumes. Our results are also in line with those reported by Xiong et al [14, 25]. However, the potential impact on patients’ selection and outcome either in the early or in the late time window remained unclear.

According to the American Heart Association [26] guidelines, no specific advanced imaging selection criteria are required for EVT in patients who present within 6 h of LPGH and have anterior circulation LVO. Nevertheless, there are proofs that perfusion imaging profiles predict the clinical response to EVT even in this early time window [3, 24] when applying the criterion of ischemic core < 70 mL and a DWI-PWI mismatch ratio > 1.2 [3]. In accordance, we found that up to 49.2% of patients having a favourable mismatch profile had functional independence at 3 months after successful EVT. However, our results also indicate that method C is more restrictive for patients’ selection, while 19.3% of patients with a good outcome would not have undergone EVT when basing selection only on imaging criterion. As all multicentre studies used method C for patients’ selection, our results may suggest that more AIS patients could in fact benefit from EVT in the early time window than previously reported. This may also imply that selection for EVT should not be only based on imaging criterion in the early time window.

In the late time window, EVT is recommended for AIS patients who present within 6 to 16 h of LPGH, have anterior circulation LVO and meet the DEFUSE 3 (infarct core < 70 mL, mismatch ratio ≥ 1.8) or DAWN (mismatch between the severity of the clinical deficit and the infarct volume) inclusion criterion. In the present study, when applying DEFUSE 3 criterion at our cohort, a larger number of patients was classified has having an unfavourable mismatch profile when using method C versus A and B. This indicates that method C could be more restrictive for selecting patients eligible for EVT. Indeed, in the late time window, 16/42 (38.1%) patients with successful EVT would not have undergone EVT with method C compared to 3/42 (7.1%) or 2/42 (4.8%) with method A or B. This is in line with Deutschmann et al [15] who reported that application of the DEFUSE 3 threshold (i.e. core < 70 mL only) would have resulted in revoking 7.4% of patients with RAPID® compared to Olea Sphere®. Interestingly, of 18 patients with functional independence at 3 months, 7/18 (38.9%) would not have been treated using method C, which suggests that patients’ outcome may be significantly influenced by the type of software used for initial imaging evaluation. Ducroux et al [27] reported that 7/17 (41%) DEFUSE 3–ineligible patients and 16/42 (38%) DAWN-ineligible patients who underwent EVT did benefit from this treatment. This could be partly due to software package as we found that 7/16 (43.8%) patients with unfavourable mismatch profile as indicated by method C had functional independence at 3 months. Similarly, Desai et al [28] reported that 30% of patients undergoing off-label EVT according to the DAWN and DEFUSE 3 criteria when assessed with method C achieved functional independence at 3 months. This might overall suggest that more AIS patients could benefit from late EVT than reported in multicentre trials using optimised perfusion software because they were underselected.

We have to acknowledge several limitations in our study. First, this is a retrospective study, even if it relies on a prospectively collected and consecutive cohort of patients, which can reduce some selection bias. Second, there is a risk of bias in a monocentric study; its advantage is the uniformity of clinical data collection following an identical protocol at 3T on a single MR scanner. Third, our imaging protocol used high-resolution 3-mm slice thickness diffusion and perfusion imaging, which could limit interpolation-related overestimation due to thicker slices, although the manufacturer of method C does recommend 5-mm slice thickness acquisition. Moreover, comparing methods A and C, all manual interferences on volume segmentation were avoided, both methods being independent from user manipulation-related bias and experience. On the contrary, method B included mask quality control and manual adjustment. However, this needed only minimal expertise and was poorly time-consuming (2 min per case). Furthermore, we found that revocation of patients with a good outcome might occur at a lower rate in the late time window using method B (5.5% versus 16.6% and 38.9% for methods A and C), which suggests that losing 2 min of time could at the end help saving functional independence in some patients. Taking into account recent development of artificial intelligence–based software, use of machine learning or more advanced deep learning technologies in perfusion tools may help in improving volume delineation, reduce inter-reader and inter-software variability and harmonise patients’ triage [29]. This was out of the scope of our work and needs to be evaluated in large prospective multicentre studies.

## Conclusion

MRI software used for DWI-PWI analysis may influence patients’ selection before EVT and impact clinical outcome either at the early and late time window. More restrictive software could deprive some AIS patients of the functional benefit of a successful endovascular thrombectomy. Quality control and manual refining should be considered when using fully automated software not to preclude functional independence of some patients.

## Abbreviations

AIS: Acute ischemic stroke
ASTRAL: Acute STroke Registry and Analysis of Lausanne
EVT: Endovascular thrombectomy
ICA: Internal carotid artery
IV tPA: Intravenous tissue plasminogen activator
LPGH: Last proof of good health
LVO: Large vessel occlusions
M1: Middle cerebral artery, horizontal segment
M2: Middle cerebral artery, insular segment
mTICI: Modified Treatment in Cerebral Ischemia score
NIHSS: National Institutes of Health Stroke Scale
PACS: Picture Archiving and Communication System
PWI: Perfusion-weighted imaging
sICHECASS: Symptomatic intracranial haemorrhage according to the criteria of the European Cooperative Acute Stroke Study
SIT-uv: Severely ischemic tissue with uncertain viability
TOAST: Trial of ORG 10172 in Acute Stroke Treatment

## Declarations

### Guarantor

The scientific guarantor of this publication is Vincent Dunet.

### Statistics and biometry

One of the authors (Vincent Dunet) has significant statistical expertise. No complex statistical methods were necessary for this paper.

### Methodology

• retrospective

• observational

• performed at one institution
